# Unveiling the Intricacies: Exploring Stepwise Initiation of Peritoneal Dialysis in a Single-Center Setting

**DOI:** 10.3390/medicina60101723

**Published:** 2024-10-21

**Authors:** Ping-Kun Hsiao, Wei-Je Wong, Su-I Hsieh, Hsiu-Ying Lin, Tzay-Jinn Chen, Chung-Yi Cheng

**Affiliations:** 1 Department of Surgery, Wan Fang Hospital, Taipei Medical University, No. 111 Sec. 3, Xinlong Rd, Taipei 11696, Taiwan; 86301@w.tmu.edu.tw; 2 Division of Nephrology, Department of Internal Medicine, Wan Fang Hospital, Taipei Medical University, No. 111 Sec. 3, Xinlong Rd, Taipei 11696, Taiwan; b101107154@tmu.edu.tw (W.-J.W.); 100456@tmu.edu.tw (T.-J.C.); 3 Department of Nursing, Wan Fang Hospital, Taipei Medical University, Taipei 11696, Taiwan; 94002@w.tmu.edu.tw (S.-I.H.); 87391@w.tmu.edu.tw (H.-Y.L.); 4 Hemodialysis Center, Wan Fang Hospital, Taipei Medical University, Taipei 11696, Taiwan; 5 Research Center of Urology and Kidney, Taipei Medical University, No. 250 Wuxing St., Taipei 11031, Taiwan; 6 Division of Nephrology, Department of Internal Medicine, School of Medicine, College of Medicine, Taipei Medical University, No. 250 Wuxing Street, Taipei 11031, Taiwan

**Keywords:** peritoneal dialysis, stepwise initiation of peritoneal dialysis, chronic kidney disease, exit-site infection, tunnel infection

## Abstract

*Background and Objectives:* Chronic kidney disease (CKD) poses a significant global health challenge, necessitating effective renal replacement therapies. Peritoneal dialysis (PD) offers a patient-friendly, home-based alternative to hemodialysis. The Stepwise Initiation of Peritoneal Dialysis (SIPD) method, used in the SPD group and involving a gradual introduction of PD, presents a potential advantage over traditional protocols, yet the scientific literature on its efficacy and safety is limited. *Materials and Methods*: We conducted a retrospective analysis of 39 end-stage renal disease patients undergoing SIPD and 78 patients receiving conventional PD (CPD) at a single center from 1 January 2010 to 31 December 2023. Patients were matched for age and sex. Surgical techniques, early and late complications, infection rates, and catheter survival were evaluated. Data were analyzed using statistical methods, including the chi-square test, *t*-test, and negative binomial regression. *Results*: The mean break-in period was significantly more extended for the SPD group (176.05 ± 154.39 days) compared to the CPD group (26.87 ± 58.45 days). Early complications were similar between groups, but late complications, including peritonitis, were significantly higher in the CPD group. The SPD group experienced fewer infection events (28 vs. 80, *p* = 0.043). Median catheter survival times were 1486 days for SPD and 1774 days for CPD, with no statistical difference. Age was a significant factor in peritonitis incidence, increasing with age in both groups. *Conclusions*: Our study suggests that SPD may reduce the incidence of catheter-related infections and peritonitis compared to CPD. The extended break-in period in SPD could enhance tissue healing and reduce biofilm formation, thereby contributing to fewer infectious complications. Despite these findings, no significant difference in overall catheter survival was observed. Further multi-center studies with larger sample sizes are recommended to confirm these results and explore the economic impact of SPD vs. CPD.

## 1. Introduction

Chronic kidney disease (CKD) remains a formidable global health challenge, affecting millions of individuals worldwide. With the escalating prevalence of CKD, there is a need for effective renal replacement therapies [1]. Peritoneal dialysis (PD) has emerged as a pivotal option among the array of modalities, providing a patient-friendly, home-based alternative to hemodialysis [2,3].

Traditionally, the initiation of PD follows a standard protocol. It is recommended that a PD catheter should be inserted a minimum of 2 weeks before the initiation of PD to allow for adequate wound healing and prevent a dialysate leak [4,5]. The Stepwise Initiation of Peritoneal Dialysis (SIPD) method represents a distinctive approach that has captured attention within the nephrology community [6]. SIPD entails a gradual and systematic introduction of PD, departing from the immediate full-volume exchanges conventionally practiced. This innovative catheter design has also been purported to enhance the quality of patient care and reduce healthcare costs [7]. SIPD reduces the early exposure of the peritoneal membrane to high dialysis volumes and solutions, potentially minimizing long-term damage or fibrosis to the peritoneal membrane.

Moreover, SIPD gradually increases the dialysis dose, allowing clinicians to closely monitor the patient’s response and adjust the therapy as needed. This incremental dialysis method can result in a gentler and more personalized approach to dialysis care. Despite its potential advantages, the scientific literature on SIPD remains sparse, leaving a substantial gap in our comprehension of its efficacy, safety, and long-term outcomes.

This study contributes to existing knowledge by conducting a detailed analysis of 39 cases involving the SIPD method. We aim to illuminate the clinical experiences, challenges, and outcomes of SIPD, providing valuable insights that may inform future clinical practice and research endeavors.

Through a comprehensive scrutiny of patient records, clinical parameters, and follow-up data, we seek to address critical questions regarding SIPD, such as its influence on peritoneal membrane adaptation, infection rates, and overall patient satisfaction. By presenting a comprehensive overview of these cases, we aspire to enhance the evidence base for SIPD and stimulate further investigations into this novel approach to peritoneal dialysis initiation.

As we embark on this exploration of SIPD, we anticipate that our findings will contribute to a refining of the peritoneal dialysis protocols and set the stage for future prospective studies to establish SIPD as a safe and efficacious alternative in managing CKD.

## 2. Materials and Methods

Following approval from the institutional review board (TMU-jointed IRB, approval No. N202404007), we conducted a thorough retrospective analysis of end-stage renal failure cases from 1 January 2010 to 31 December 2023. Patients undergoing Stepwise Initiation of Peritoneal Dialysis (SIPD) comprised Group SPD, while those undergoing conventional peritoneal dialysis catheter insertion constituted Group CPD. Demographic information and relevant clinical data were meticulously extracted from electronic medical records. Both groups were rigorously matched based on age and sex to ensure comparability, with a ratio of SPD to CPD of 1:2.

### 2.1. Surgical Techniques

All patients underwent PD catheter insertions via laparoscopic surgery under general anesthesia. A straight Tenckhoff catheter was used in our institution. The intraperitoneal end was gently affixed to the peritoneum of the anterior abdominal wall using sutures and directed into the pelvis. Subsequently, the proximal end of the catheter was tunneled subcutaneously using a trocar from the insertion site to a predetermined marked exit point. All catheters underwent confirmation of acceptable flow functions, were flushed with heparin, and then securely plugged. Following these procedures, the catheters were either buried in the subcutaneous tissue before skin closure (SPD group) or left externalized (CPD group).

**Figure 1:**
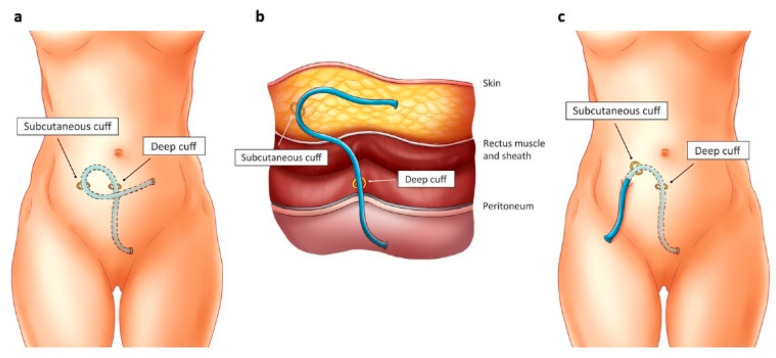
(**a**). A laparoscopically inserted buried Tenckhoff catheter. (**b**). Illustration of the position of the subcutaneous and deep cuffs. (**c**). A laparoscopically inserted catheter ready for use.

Buried catheters remained embedded until the initiation of renal replacement therapy. The same surgeon conducted exteriorization in an outpatient setting during the patient’s initial PD training session in preparation for dialysis. The catheter’s superficial cuff and distal subcutaneous end were identified through palpation, and a skin marking was made accordingly. Subsequently, under aseptic conditions, a small transverse incision was made approximately 2 cm distal to the superficial cuff, and the catheter was retrieved using forceps. Once exteriorized, the catheter was clamped, fitted with an adaptor, attached to a transfer set, and thoroughly flushed with saline to eliminate fibrin plugs. To enhance understanding of the surgical technique, we have illustrated it in Figure 1a–c.

### 2.2. Patient Satisfaction Survey

We used a six-question Likert scale (1–5) questionnaire to measure patient satisfaction within the SPD group. The questionnaire is attached to the Supplementary File. We sent out 21 questionnaires, and all patients responded.

### 2.3. Statistical Analysis

All data were summarized and displayed as mean values ± standard deviations (SDs) for the continuous variables, the number of patients, and the percentage in each group for categorical variables. The chi-square statistic was used to assess the statistical significance between the groups for all categorical variables. Continuous variables were first tested for normal distribution using the Kolmogorov–Smirnov test and quantile–quantile plots; then, the parameters were compared by using a *t*-test if normally distributed or by the Kruskal–Wallis/Mann–Whitney U test if not normally distributed.

## 3. Results

### 3.1. Baseline Characteristics and Break-In Period

The mean break-in period for the SPD group was 176.05 ± 154.39 days (*n* = 39), and 26.87 ± 58.45 days (*n* = 78) in the CPD group. CKD was primarily associated with diabetes mellitus (DM) in both groups, accounting for 38.5% and 47.4% in the SPD and CPD groups, respectively. Other causes of CKD and the baseline characteristics are detailed in Table 1.

### 3.2. Early and Late Complications After the Start of PD

Early complications are arbitrarily defined as catheter-related complications that develop within one month of PD initiation, while late complications develop after one month of PD initiation. Although more early complications were observed in the CPD group compared to the SPD group, no statistical significance was found. However, a significant difference was noted in the late peritonitis rate and total late complications within the CPD group (Table 2).

### 3.3. Infection Event and Catheter Survival

**Figure 2:**
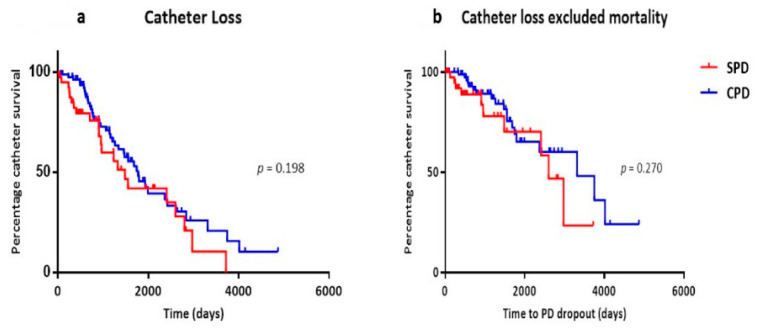
Catheter survival in the SPD and CPD groups. (**a**) Catheter loss in all patients, including mortality as a cause of catheter loss. (**b**) Catheter loss excluding patients where mortality was the cause.

PD-associated infections encompass exit-site infections (ESI), tunnel infections, and peritonitis. Occurrences of infection were stratified according to different time sequences (Table 3). A significant statistical disparity was detected in the composite infection events between the two groups (28 vs. 80 events, *p* = 0.043). Catheter survivals were assessed from the initiation of PD until the removal of the PD catheter or death. The median time for catheter survival was 1486 days for the SPD group and 1774 days for the CPD group. However, no statistical significance was observed despite the SPD group’s shorter catheter survival time. When excluding patients with mortality as the cause of PD discontinuation, there were 10 catheter losses in the SPD group and 19 in the CPD group. Again, no statistical difference was noted (Figure 2).

### 3.4. Risk of Infection Stratified to DM and All-Cause Mortality

Several factors may influence the potential causes of infection. Patients who died before developing an infection might have affected the analysis of possible infection causes. Therefore, patients who died without an infection were excluded from the study. There was no statistically significant difference between patients with diabetes mellitus (DM) or mortality in the occurrence of infection complications (Table 4a).

### 3.5. The Causative Organisms as the First Peritonitis

The causative organisms of the first episode of peritonitis were categorized into three groups. Group I included *Staphylococcus, Acinetobacter, Corynebacterium, and Streptococcus* species. Group II included *Escherichia, Klebsiella, and Enterococcus* species. A case infected by the non-tuberculosis mycobacterium was also categorized in group II. In group III, no organisms were cultured (Table 4b).

### 3.6. Predictive Risk of Peritonitis in SPD and CPD Groups

The probability of catheter survival was similar between the groups throughout the study period, commencing with PD initiation and continuing until 31 December 2023 (*p* = 0.270). Negative binomial regression analysis indicated a mean peritonitis incidence of one episode at 21.8 months and 19.9 months in the SPD and CPD groups, respectively. The subject-specific peritonitis incidence was Log t − 7.931 + 0.0229 × age, where t represents the follow-up time in days [8]. Based on this formula, a 10-year increase in patient age brought a 2.25- and 2.55-fold increase in the incidence of peritonitis in the SPD and CPD groups, respectively, (*p* = 0.023).

## 4. Discussion

Our study observed a trend suggesting a decreased incidence of catheter-related infections when utilizing embedded PD catheters compared to non-embedded catheters. Patients with embedded catheters (SPD group) exhibited a significantly lower likelihood of experiencing catheter-related infections and peritonitis over time than those with non-embedded catheters (CPD group). Moreover, the interval between the initiation of PD and the onset of the first catheter-related infection was notably prolonged in the SPD group. This reduced risk of infectious complications associated with embedded catheters may be attributed to enhanced tissue ingrowth around the superficial cuff due to an extended healing period of the peritoneal wall, thereby inhibiting biofilm formation [9]. Furthermore, our investigation revealed similar rates of complications, such as hernia, leakage, hydrothorax, and outflow failure, between individuals receiving embedded and non-embedded catheters.

Currently, the literature comparing catheter-related complications between stepwise initiation of peritoneal dialysis (SIPD) and the conventional laparoscopic Tenckhoff catheter insertion is limited and yields conflicting results. Most published data are based on the modified Moncrief and Popovich technique via open surgery [8,10]. Our study’s findings diverge from those of a prospective randomized study by Danielsson et al., which involved 60 participants and found no reduction in ESIs or peritonitis rates with buried catheters before initiating peritoneal dialysis (PD) [11]. In contrast, a prospective study by Keskar et al. reported an increased incidence of ESIs in the buried catheter group compared to the non-buried group [12]. Similarly, a prospective study by Park et al. found a significantly reduced incidence of catheter-related peritonitis in patients with buried catheters compared to those using the conventional technique [13]. Additionally, Tan et al. reported a higher proportion of infections in the non-buried catheter group compared to the buried catheter group [14]. While the exact mechanism by which pre-embedded catheters prevent peritonitis is not fully understood, it is believed to be related to the inhibition of biofilm formation. However, the precise molecular mechanism remains unknown [9].

We observed no statistical differences in catheter survival between the SPD and CPD groups. This finding is consistent with a more extensive series reported by Wu et al. [8], which also found similar catheter survival and peritonitis rates in patients with buried and non-buried catheters. Additionally, when accounting for mortality as a cause of PD dropout, we still found no difference in catheter survival between the two groups.

The buried catheter offers a key advantage due to its elective nature, enhancing both patient health and convenience. The increased flexibility in the timing for insertion reduces the risk of requiring hemodialysis during a break-in period if renal function unexpectedly declines [15]. In our study, none of the patients in the SPD group required hemodialysis during the break-in period. The decision on catheter insertion was made at the physician’s discretion. Additionally, our survey showed a relatively high satisfaction rate among SPD patients, with an average score of 4.25 out of 5 on a Likert scale (Supplementary Materials Table S1). We also analyzed the baseline and latest Kt/V measurements in patients with and without peritonitis and found no statistically significant differences (1.83 ± 0.51 vs. 2.04 ± 0.32, peritonitis vs. no peritonitis, respectively, in the SPD group, *p* = 0.584). The results are shown in the Supplementary materials as Figure S1.

Several limitations are present in our study. It represents a single-center experience reporting the outcomes of a single surgeon, and despite identifying a significant difference in the peritonitis rate between the two groups, our sample size was relatively small. A larger sample size is necessary to strengthen the power of one technique over another. Further research involving multiple centers comparing the infectious and mechanical complications of various insertion techniques (e.g., buried vs. non-buried laparoscopic techniques) and the economic cost to the healthcare system is recommended.

## 5. Conclusions

Our study indicates that SPD may lower the incidence of catheter-related infections and peritonitis compared to CPD. The more extended break-in period in SPD may improve tissue healing and decrease biofilm formation, potentially leading to fewer infectious complications. However, no significant difference in overall catheter survival was found. We recommend further multi-center studies with larger sample sizes to confirm these findings and investigate SPD’s economic implications compared to CPD patients.

## Figures and Tables

**Table 1:** Baseline characteristics of SIPD vs. conventional catheter insertion groups.

	SPD (*n* = 39)	CPD (*n* = 78)	*p*-Value
Age (yrs)	61 (22)	58 (18.25)	0.265
Sex (m, %)	18 (46.2%)	40 (51.3%)	0.696
BW (kg)	65 (22.5)	64.7 (19.2)	0.429
BMI (kg/m^2^)	24.8 (7.05)	24.7 (5.45)	0.443
Kt/V (IQR)	2.0 (0.44)	1.9 (0.25)	0.039 *
WCC (IQR)	65.91 (30.39)	60.59 (26.68)	0.231
Hemoglobin (g/dL)	9.1 (2.10)	8.7 (1.55)	0.097
Platelet (×10^3^/μL)	173 (204.79)	207 (80.50)	0.121
Albumin (g/dL)	3.8 (0.50)	3.6 (0.63)	0.080
Cause of Renal Failure			
DM Nephropathy	15 (38.5%)	38 (48.7%)	0.239
HTN nephrosclerosis	14 (35.9%)	22 (28.2%)	0.396
PCKD	3 (0.76%)	1 (0.13%)	0.113
Glomerulonephritis	5 (12.8%)	14 (17.9%)	0.480
Others	2 (0.05%)	3 (0.04%)	0.747

Abbreviations: BW, body weight; BMI, body mass index; WCC, weekly creatinine clearance; HTN, hypertension. * *p* < 0.05.

**Table 2:** Early and late complication rates of SIPD and conventional catheter insertion groups.

	SPD (*n* = 39)	CPD (*n* = 78)	*p*-Value
Early (<1 month)			
Bowel perforation	0	0	-
Bleeding	0	0	-
Outflow failure	0	0	-
Leakage	0	1 (1.28%)	0.999
Exit-site infection	2 (5.13%)	3 (3.85%)	0.747
Tunnel infection	0	0	0
Peritonitis	0	5 (6.41%)	0.999
Hydrothorax	0	0	0
Total early complications	2 (5.13%)	8 (10.26%)	0.359
Late (>1 month)			
Exit-site infection	3 (7.69%)	10 (12.82%)	0.410
Tunnel infection	1 (2.56%)	3 (3.85%)	0.721
Peritonitis	12 (30.77%)	42 (53.84%)	0.014 *
Leakage (>1 month)	0	2 (2.56%)	0.999
Hernia	4 (10.26%)	10 (12.82%)	0.688
Hydrothorax	0	2 (2.56%)	0.999
Total Late complications	20 (51.28%)	70 (92.3%)	0.016 *
All-cause mortality	12 (30.77%)	22 (28.21%)	0.773

* *p* < 0.05.

**Table 3:** Infection complications stratified according to the timing of infection.

Time to Occurrence of Complication	SPD (*n* = 39)	CPD (*n* = 78)	*p*-Value
≤1 month			
Exit-site infection	2 (5.13%)	3 (3.85%)	0.747
Tunnel infection	0	0	-
Peritonitis	0	5 (6.41%)	0.999
1–3 months			
Exit-site infection	0	1 (1.28%)	1.000
Tunnel infection	0	0	-
Peritonitis	1 (2.56%)	4 (5.13%)	0.526
3–6 months			
Exit-site infection	0	2 (2.56%)	0.999
Tunnel infection	0	0	-
Peritonitis	1 (2.56%)	10 (12.82%)	0.107
6 months–1 year			
Exit-site infection	2 (5.13%)	6 (7.69%)	0.607
Tunnel infection	0	1 (1.28%)	1.000
Peritonitis	1 (2.56%)	6 (7.69%)	0.294
≥1 year			
Exit-site infection	11 (28.21%)	15 (19.23%)	0.274
Tunnel infection	2 (5.13%)	2 (2.56%)	0.480
Peritonitis	8 (20.51%)	25 (32.05%)	0.195
Composite Infection Complications	28	80	0.043 *

* *p* < 0.05.

**Table 4:** (a) Risk of infection stratified to DM and all-cause mortality in SIPD and conventional catheter insertion groups. (b) The causative organisms are the first peritonitis.

**(a)**										
**Variable**	**Estimate**		**SE**		***p*** **Value**	**OR**		**Lower CI**		**Upper CI**
DM	0.112		0.394		0.776	1.118		0.517		2.420
Mortality	0.549		0.329		0.095	1.731		0.909		3.297
**(b)**										
		**Group 1**		**Group 2**			**Group 3**		**Total**	
SPD		4 (33.3%)		8 (66.7%)			0		12	
CPD		22 (52.4%)		6 (14.3%)			14 (33.3%)		42	

## Data Availability

The datasets generated during and/or analyzed during the current study are available from the corresponding author on reasonable request.

## Supplementary Materials

The following supporting information can be downloaded at: https://www.mdpi.com/article/10.3390/medicina60101723/s1, Table S1: Patient Satisfaction Survey for SIPD. Figure S1: The patients’ Kt/V values in the SPD group without peritonitis vs. patients with peritonitis. No statistical significance was noted.
