# Supplementary material for: Unveiling the Intricacies: Exploring Stepwise Initiation of Peritoneal Dialysis in a Single-Center Setting
Source: Medicina (Kaunas). 2024 Oct 21;60(10):1723. doi: 10.3390/medicina60101723 (PMC11509374; doi:10.3390/medicina60101723)
Supplement: Supplementary file 1 [file medicina-60-01723-s001.zip › medicina-3213173-supplementary.pdf]

Supplementary Table S1. Patient Satisfaction Survey for SIPD

Question 1	Do you feel that the two-stage peritoneal dialysis method is acceptable to you?
Do not know	0
Not acceptable	1
Slightly unacceptable	1
Partially acceptable	5
Completely acceptable	14
Average	4.52

Question 2	Regarding the two-stage peritoneal dialysis, with the second surgery required to pull out the dialysis catheter, do you find it troublesome when you think about it now?
Do not know	0
Very troublesome	4
Slightly troublesome	3
It is acceptable	9
Not troublesome at all	5
Average	3.71

Question 3	I chose the two-stage peritoneal dialysis, which gives me buffer time to arrange for my long-term dialysis treatment.
Do not know	0
Disagree	1
Partially disagree	2
Partially agree	2
Completely agree	16
Average	4.57

Question 4	If you must undergo dialysis treatment and could choose again, would you still opt for the two-stage peritoneal dialysis?
Do not know	0
Definitely not	1
Probably not	2
Probably yes	8
Definitely yes	10
Average	4.29

Question 5	If any of your relatives or friends are facing the need for dialysis, would you recommend the two-stage peritoneal dialysis for their consideration?
Do not know	0

Definitely not<br>Probably not<br>Probably yes<br>Definitely yes	2<br>1<br>9<br>9
Average	4.05

Question 6	So far, has your experience with the two-stage peritoneal dialysis met the expectations of your initial decision?
Do not know<br>Completely not met expectations<br>Partially not met expectations<br>Partially met expectations<br>Completely met expectations	0<br>0<br>4<br>6<br>11
Average	4.33

Total average	4.25

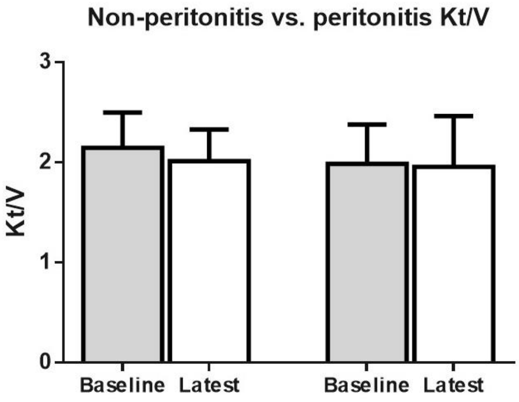

Figure S1. The patients' Kt/V values in the SPD group without peritonitis vs. patients with peritonitis. No statistical significance was noted.
